# Fracture des épines tibiales chez l’enfant

**DOI:** 10.11604/pamj.2017.28.244.11304

**Published:** 2017-11-17

**Authors:** Abdoulaye Diallo Harouna, Hind Cherrabi, Karima Atarraf, Lamiae Chater, Abderrahmane My Afifi

**Affiliations:** 1 Service de Traumato-Orthopédique Pédiatrique, CHU Hassan II, Fès, Maroc; 2 Université Sidi Mohamed Ben Abdellah, Faculté de Médecine et de Pharmacie de Fès, Maroc

**Keywords:** Fracture de l´épine tibiale, accident du sport, enfant, Tibial spine fracture, sport accident, child

## Abstract

La fracture des épines tibiales est une lésion rare qui survient généralement chez les adolescents sportifs entre 8 et 17 ans. Le traitement des formes déplacées nécessite une réduction chirurgicale et une fixation afin d’offrir au ligament croisé antérieur une tension adéquate et réduire le risque de laxité. Le but de cette étude était de rapporter notre expérience dans la prise en charge des fractures de l’épine tibiale chez l’enfant. Il s’agit d’une étude rétrospective colligée sur une période de 7 ans (2009-2016) intéressant 11 cas de fractures de l’épine tibiale chez l’enfant. L’âge moyen de nos malades était de 13 ans et demi. La chute lors du sport était en cause dans 73% des cas. La classification de Meyers et Mac Keever, modifiée par Zaricznyj a été adoptée, elle a permis de classer les lésions en 4 types. Deux cas ont été traités orthopédiquement et 9 cas ont bénéficié d’une réduction chirurgicale par arthrotomie et une fixation par ostéo-suture. Avec un recul moyen de 3 ans, nos résultats sont jugés bons dans 91% selon le score fonctionnel de Lysholm. Un seul cas de type II traité orthopédiquement a présenté un score fonctionnel de Lysholm jugé encore moyen. Afin d’assurer un bon tonus au ligament croisé antérieur, en dehors du type I, il nous semble défendable d’opter de façon systématique pour un traitement chirurgical pour les types II à IV. La fracture des épines tibiales est de bon pronostic. La réduction chirurgicale est la règle à chaque fois qu’un déplacement s’y associe afin de mieux vérifier l’intégrité du ligament croisé antérieur et de garantir une bonne stabilité genou.

## Abstract (en)

Tibial spine fractures are rare lesions which usually occur in adolescent athletes aged between 8 and 17 years. The treatment of displaced fractures requires surgical reduction and fixation in order to achieve adequate tension in the anterior cruciate ligament and to reduce the risk of laxity. This study aimed to report our experience in the treatment of tibial spine fractures in children. We conducted a retrospective study of 11 children with tibial spine fractures over a period of 7 years (2009-2016). The average age of our patients was 13 years and a half. A fall during sport was the main cause of tibial spine fractures in 73% of patients. Meyers and Mac Keever classification, modified by Zaricznyj, was used and allowed to classify the lesions into 4 types. Two cases were orthopedically treated while 9 cases underwent surgical reduction through arthrotomy and fixation using osteosuture. After an average follow-up of 3 years our results were good in 91% of cases, according to Lysholm functional score. Only one case with orthopedically treated type II fracture still had a mean Lysholm functional score. Surgical treatment for types II-IV fractures (with the exception of type I) should be used in order to ensure a good anterior cruciate ligament tonus. The prognosis of tibial spine fractures is good. Surgical reduction should be used when tibial spine fractures are associated with displacement in order to better verify anterior cruciate ligament integrity and to ensure a good knee stability.

## Introduction

La fracture des épines tibiales est relativement rare, son incidence est de 3 cas/100.000 fractures de l’enfant, les adolescents sportifs entre 8 et 17 ans étant plus exposés [1-3]. La classification radiologique de Meyers et Mac Keever, modifiée par Zaricznyj a permis de repartir les lésions en 4 types [4]. Si le type I répond au traitement orthopédique, les autres types nécessitent généralement une réduction chirurgicale [1,2,5]. L’objectif de cette étude est d’analyser nos résultats à la lumière de la littérature.

## Méthodes

De Janvier 2009 à Janvier 2016 nous avons revu rétrospectivement 11 dossiers de patients traités pour fracture des épines tibiales. Sont inclus dans cette étude, tous patients dont l’âge est inférieur ou égale à 16 ans et traités dans le service, avec un recul minimum de 6 mois. Nous avons éliminé tous les patients traités initialement dans d’autres services et les patients perdus de vue. 11 patients ont répondu à nos critères dont 7 garcons et 4 filles. La classification de Meyers et Mac Keever, modifiée par Zaricznyj a été adoptée dans notre série. Nos résultats ont été évalués selon le score fonctionnel de Lysholm et la qualité de la réduction sur la radiographie standard.

## Résultats

**Figure 1:**
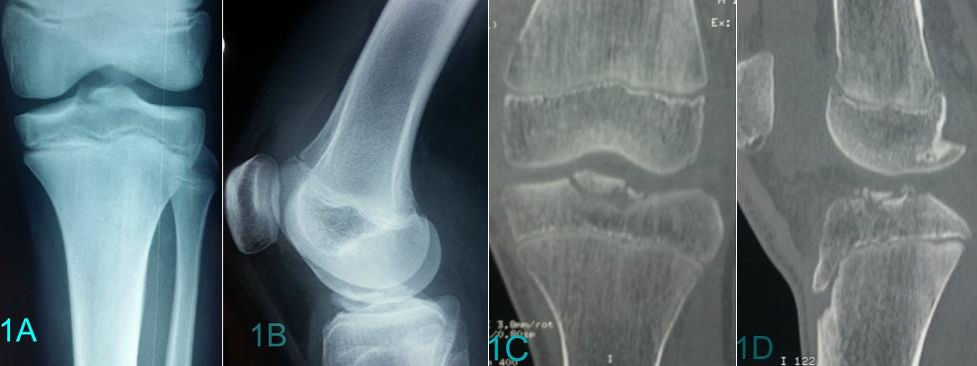
Cliché radiographique de face (A) et de profil (B) montrant une fracture des épines tibiales; (C, D) images tomodensitométriques avec reconstruction montrant la fracture des épines tibiales

**Figure 2:**
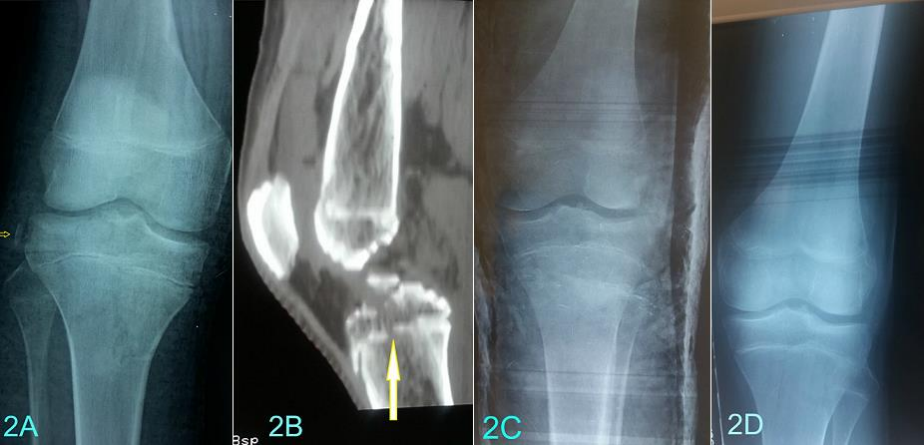
A) arrachement du ligament latéral externe (flèche jaune); B) suspicion d’une lésion méniscale à la tomodensitométrie (flèche); C) fracture type traitée orthopédiquement avec bonne consolidation après déplâtrage (D)

**Figure 3:**
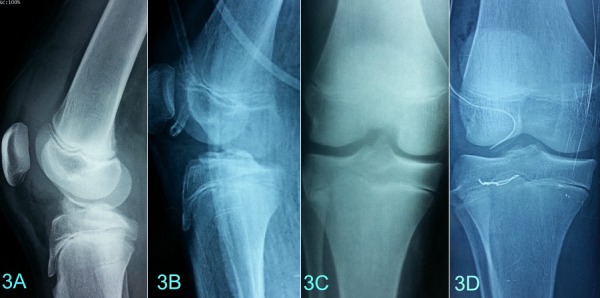
Fracture type III avant (A) et après ostéo-suture au fil résorbable (B); (C, D) fractures type III avant (C) et après suture au fil d’acier (D)

L’âge moyen était de 13 ans et demi (extrêmes: 8 ans et 15 ans et demi). Les accidents lors du sport étaient en cause dans 80%. Un seul patient était victime d’un accident de la voie publique. L’examen clinique trouvait une hémarthrose, une douleur à la mobilisation du genou et une impotence fonctionnelle chez 10 patients. La radiographie standard du genou de face et de profil a permis de poser le diagnostic dans 73% des cas (Figure 1 (A et B)), une tomodensitométrie du genou a été nécessaire chez 3 patients présentant un doute diagnostique (Figure 1 (C et D)). Deux cas de lésions associées ont été suspectés respectivement, une lésion méniscale et un arrachement du ligament latéral externe (Figure 2 (A et B). Les fractures étaient reparties comme suite: un cas de type I, cinq cas de type II, quatre cas de type III et un cas de type IV. Le délai moyen de prise en charge était de 3jours. Deux cas ont été traités orthopédiquement dont 1 cas de type I et 1 cas de type II (Figure 2(C et D)) et 9 cas ont bénéficié d’une réduction chirurgicale. La voie d’abord était une arthrotomie para-patellaire externe, l’exploration avait confirmé la présence des lésions suspectées radiologiquement. Le traitement chirurgical a consisté à une réduction avec une fixation par du fil résorbable dans 8 cas (Figure 3(A et B)), et par du fil d’acier dans un cas (Figure 3 (C et D)), les lésions associées étaient traitées en même temps. Une immobilisation en extension par un plâtre curo-pédieux a été indiquée dans tous les cas pendant en moyenne 45 jours. Avec un recul moyen de 3 ans (extrêmes; 6 mois et 7 ans). Le score fonctionnel de Lysholm au déplâtrage était de 82 en moyenne (extrêmes: 69 et 90) au dernier recul et de 98,2 (extrêmes: 80 et 100). Au dernier recul, 91% des patients ont repris leurs activités habituelles et sportives sans gêne fonctionnelle, un seul patient présentait encore une laxité au test de Lachmanet trois (3) patients avaient présenté une raideur du genou ayant régressé après un mois de rééducation.

## Discussion

La fracture des épines tibiales est relativement rare, 3 cas sur 100.000 fractures de l’enfant [1], elle survient habituellement chez les adolescents (entre 8 et 17 ans) sportifs [2, 6,7]. L’âge moyen de nos patients était de 13 ans et demi, et rejoint celui retrouvé dans la littérature (extrêmes 8 ans et 15ans et demi) les accidents du sport étaient en cause dans 80% des cas. L’arrachement de l’épine tibiale est l’équivalence lésionnelle de la rupture du ligament croisé antérieur chez l’adulte. En effet, chez l’enfant, l’épine tibiale n’est pas encore complètement ossifiée pour cet effet, les fibres ligamentaires sont en continuité avec le périchondre du cartilage de conjugaison, ainsi tout traumatisme mettant en tension ce ligament croisé antérieur expose inéluctablement à un arrachement de l’épine tibial chez l’enfant [1,8]. La présence à l’examen clinique d’un gros genou douloureux post-traumatique doit faire évoquer le diagnostic et conduit à la réalisation d’un bilan radiologique du genou [3,9]. La radiographie standard du genou en double incidence de face et de profil pose généralement le diagnostic [5]. Une tomodensitométrie voire l’imagerie par résonnance magnétique sont demandées en cas de doute diagnostique afin d’établir un bilan lésionnel précis [2].

Dans notre série, un complément tomodensitométrique n’a été nécessaire que dans 3cas. La plupart des auteurs s’accorde à traiter orthopédiquement les fractures de type I et un traitement chirurgical est préconisé pour les types III et IV [2,3,5]. Cependant les avis restent encore partagés quant à la prise en charge du type II, pendant que Casalonga et al. [5] adoptent un traitement orthopédique pour les types II peu déplacés, l’équipe de Louis ML [6] préfère une réduction chirurgicale d’emblée. Afin de mieux assurer au ligament croisé antérieur un tonus optimal, notre stratégie thérapeutique rejoint celle de l’équipe de Louis ML à savoir un traitement orthopédique pour tous les types I et chirurgical pour le reste. Dans notre série 2 patients ont bénéficié d’un traitement orthopédique sans réduction préalable, dont un cas du type II, il avait un traumatisme crânien associé nécessitant une surveillance en milieu de réanimation. La voie d’abord était une arthrotomie para-patellaire externe.

Plusieurs moyens d’ostéosynthèses ont été décrits à savoir l’ostéo-suture par du fils résorbable, le fil d’acier et le vissage [6,9], dans notre série le fils résorbable a été largement utilisé chez 8 patients et le fil d’acier dans 1 cas. La réduction chirurgicale peut également être menée sous arthroscopie avec les mêmes principes de base d’ostéosynthèse que l’arthrotomie [9,10]. Au dernier recul, nous avons retrouvé un score fonctionnel moyen de Lysholm à 98,2 nettement meilleur par rapport à celui de Jarnav et al qui était de 94,3 [11], mais reste inférieur au score de l’équipe de Louis ML qui n’étudiait que le type II [5]. Nous n’avons pas retrouvé de lien entre la qualité de la réduction sur la radiographie et le résultat fonctionnel. En effet, parmi les 4 patients de notre série qui présentaient un défaut de réduction, un seul cas de gêne fonctionnelle a été retrouvé. Bien que le recul moyen (3 ans) soit très faible dans notre série, la laxité résiduelle habituellement rapportée aussi bien après le traitement orthopédique que chirurgical n’a été retrouvé que chez un cas de type II qui a été traité orthopédiquement [12,13]. Cette laxité résiduelle semble probablement liée à une déformation plastique du ligament croisé antérieur comme le suggèrent Noyes et al [14].

Au vu de tout ce qui précède nous estimons qu’en dehors du type I, il est défendable de préconiser un traitement chirurgical pour les types II à IV afin d’offrir au ligament croisé un bon tonus. Plusieurs techniques sont utilisées dans la réduction chirurgicale, nous pensons que la meilleure est celle que l’on maitrise.

## Conclusion

La fracture des épines tibiales reste de bon pronostic. La réduction chirurgicale est la règle à chaque fois qu’un déplacement s’y associe afin de mieux vérifier l’intégrité du ligament croisé antérieur et de garantir une bonne stabilité genou.

### Etat des connaissances actuelles sur le sujet

- Accident de sport de l’adolescent;
- Le traitement du type II ne fait pas de consensus.

### Contribution de notre étude à la connaissance

- Nous n’avons pas retrouvé de corrélation entre la qualité de la réduction sur la radiographie et le résultat fonctionnel;
- La fracture type doit obéir à une réduction chirurgicale.

## Conflits d’intérêts

Les auteurs ne déclarent aucun conflit d'intérêts.
